# Potential of Using Infrapatellar–Fat–Pad–Derived Mesenchymal Stem Cells for Therapy in Degenerative Arthritis: Chondrogenesis, Exosomes, and Transcription Regulation

**DOI:** 10.3390/biom12030386

**Published:** 2022-03-01

**Authors:** Hsiu-Jung Liao, Chih-Hung Chang, Chi-Ying F. Huang, Hui-Ting Chen

**Affiliations:** 1 Department of Orthopedic Surgery, Far Eastern Memorial Hospital, New Taipei City 220216, Taiwan; liaohsiujung@gmail.com; 2 Graduate School of Biotechnology and Bioengineering, Yuan Ze University, Taoyuan City 320315, Taiwan; 3 Department of Biotechnology and Laboratory Science in Medicine, National Yang Ming Chiao Tung University, Taipei 112304, Taiwan; bpshuang@gmail.com; 4 Institute of Biopharmaceutical Sciences, National Yang Ming Chiao Tung University, Taipei 112304, Taiwan; 5 Department of Pharmacy, School of Pharmaceutical Sciences, National Yang Ming Chiao Tung University, Taipei 112304, Taiwan; 6 Department of Fragrance and Cosmetic Science, Kaohsiung Medical University, Kaohsiung 807378, Taiwan; 7 School of Pharmacy, College of Pharmacy, Kaohsiung Medical University, Kaohsiung 807378, Taiwan

**Keywords:** IPFP-MSC, osteoarthritis, chondrogenesis, exosome, transcription regulation

## Abstract

Infrapatellar fat pad–derived mesenchymal stem cells (IPFP-MSCs) are a type of adipose-derived stem cell (ADSC). They potentially contribute to cartilage regeneration and modulation of the immune microenvironment in patients with osteoarthritis (OA). The ability of IPFP-MSCs to increase chondrogenic capacity has been reported to be greater, less age dependent, and less affected by inflammatory changes than that of other MSCs. Transcription-regulatory factors strictly regulate the cartilage differentiation of MSCs. However, few studies have explored the effect of transcriptional factors on IPFP-MSC-based neocartilage formation, cartilage engineering, and tissue functionality during and after chondrogenesis. Instead of intact MSCs, MSC-derived extracellular vesicles could be used for the treatment of OA. Furthermore, exosomes are increasingly being considered the principal therapeutic agent in MSC secretions that is responsible for the regenerative and immunomodulatory functions of MSCs in cartilage repair. The present study provides an overview of advancements in enhancement strategies for IPFP-MSC chondrogenic differentiation, including the effects of transcriptional factors, the modulation of released exosomes, delivery mechanisms for MSCs, and ethical and regulatory points concerning the development of MSC products. This review will contribute to the understanding of the IPFP-MSC chondrogenic differentiation process and enable the improvement of IPFP-MSC-based cartilage tissue engineering.

## 1. Introduction

Osteoarthritis (OA) is the most common degenerative joint disease and is characterized by progressive articular-cartilage loss. Several strategies for identifying an abundant and suitable cell population for replenishing injured hyaline cartilage through stem-cell-based cartilage-repair therapies have been explored [1,2]. Mesenchymal stem cells (MSCs) exert chondroprotective effects and differentiate into cartilage cells, and they have become an attractive cell source for cell therapy for cartilage repair. They are capable of chondrogenic induction, differentiating into chondrocytes and forming hyaline-like cartilage. They contribute to histopathological improvement by promoting cartilage regeneration [3]. Some transcription factors play an essential role in regulating the expression of gene markers of chondrocytes, including collagen types II, IX, X, and XI; aggrecan; and cartilage link proteins [4,5]. During OA progression, changes in chondrocyte function and phenotype occur due to the intracellular and extracellular signals that each chondrocyte receives and integrates into gene expression over time.

**Figure 1:**
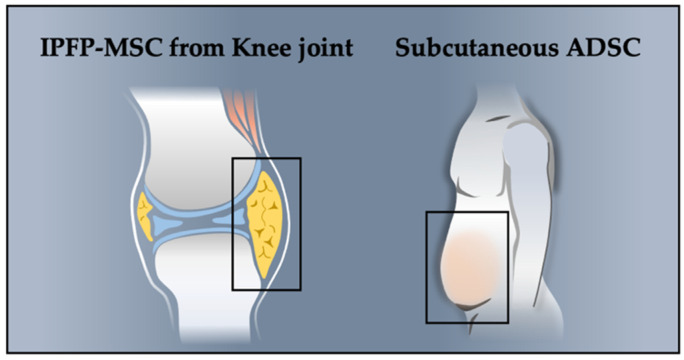
Primary sources of adipose-derived stem cells (ADSCs) in treating OA: infrapatellar fat pad–derived mesenchymal stem cells (IPFP-MSCs) and subcutaneous ADSCs.

Adipose-derived stem cells (ADSCs) are used to treat articular-cartilage injury because they are easy to harvest and exhibit high cartilaginous production capacity. ADSC sources, namely the infrapatellar fat pad (IPFP) and subcutaneous fat (SC), serve as reservoirs of MSCs. The IPFP is located in the extrasynovial area of the anterior compartment of the knee joint and is a rich source of MSCs. A large amount of adipose tissue can also be obtained from subcutaneous fat removed through liposuction (Figure 1).

Patient age is a crucial parameter that sometimes limits the success of cell therapy. Unlike those of BMSCs, the proliferation and differentiation capacities of infrapatellar fat pad–derived MSCs (IPFP-MSCs) do not differ significantly among patient age groups. This makes them promising for use in stem-cell-based regenerative therapy [6]. However, few studies have investigated transcription-factor regulation and microRNAs (miRNAs) involved in cartilage regeneration and identified the critical regulatory networks for IPFP-MSCs. In this paper, we review transcriptional factors and exosomal miRNAs from ADSCs from different sources, which may have major implications for the therapeutic potential of IPFP-MSCs and facilitate or inspire future IPFP-MSC studies.

Advanced age may inhibit the repair capacity of MSCs and may be a risk factor for OA. MSCs at late passages exhibit senescent phenotypes with reduced proliferative capacity and suboptimal multidifferentiation potential [7]. ADSC-derived exosomes possess chondroprotective and anti-inflammatory properties [8,9]. Exosomes encapsulate various molecular constituents that mediate communication among different cells and modulate multiple biological processes, including immune responses and cell differentiation [10,11]. Thus, an extracellular vesicle (EV)–mediated delivery system could be effective and efficient for the treatment of OA because it can rejuvenate senescent MSCs and can be realized using EVs from super donors. Therefore, exosomes might enable cell-free therapy, providing the same clinical benefits without the potential risks associated with infusions of living cells, such as immune rejection or tumor formation [12]. In addition, the small size and low complexity of exosomes make their production and storage easier than those for cellular methods [13].

In this paper, we first summarize the transcription factors that regulate the chondrogenesis of MSCs and exosome modulation in immune responses and evaluate the potential of using MSCs for cartilage regeneration in patients with OA. Moreover, the potential of MSC-derived-exosome cell-free therapy for OA treatment is discussed.

## 2. Positive Transcriptional Control of Chondrogenesis

Chondrogenesis is a strictly regulated multistep process mediated by chondrogenic genes and transcription factors. In this section, the essential transcription factors and related chondrogenic target genes during chondrocyte differentiation are introduced, and their roles in chondrogenesis are described. During chondrogenesis, mesenchymal cells condense and differentiate into early stage chondrocytes. Proliferating chondrocytes can produce sufficient chondrocyte-specific extracellular matrix (ECM) proteins, such as type II collagen (encoded by *Col2a1*) and aggrecan (encoded by *Acan*) [14]. Proliferating chondrocytes grow and differentiate into prehypertrophic and hypertrophic chondrocytes [15], which are characterized by the expression of Indian hedgehog (*Ihh*) and type X collagen (*Col10a1*), respectively [16,17]. These late-stage chondrocytes subsequently undergo terminal differentiation and produce matrix metalloproteinase 13 (MMP13), enabling the vascular invasion of cartilage [18]. Finally, terminal chondrocytes become apoptotic and are replaced by bone [14].

**Figure 2:**
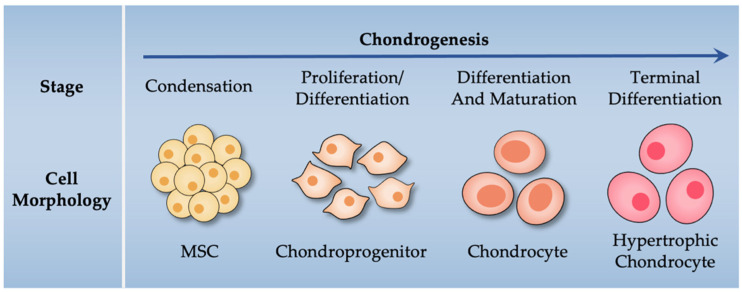
Key stages of chondrogenic differentiation of MSCs.

In Figure 2, the stages of MSCs committed to the chondroprogenitor stage of chondrocyte differentiation are illustrated. The chondrogenic differentiation of MSCs is a tightly regulated, multistep process mediated by several transcription factors, including sex-determining region Y–related high-mobility group box (SOX) proteins, trichorhinophalangeal syndrome type 1 (TRPS1), and runt-related transcription factors (RUNXs).

### 2.1. Sex-Determining Region Y–Related High-Mobility Group Box 9 (SOX9)

Genes of the SOX family, especially *SOX9*, *SOX6*, and *SOX5*, are essential for chondrogenesis [5]. *SOX9* can induce the differentiation of MSCs into chondrogenic lineages. *SOX5* and *SOX6* are similar in structure, and their functions are redundant in chondrogenesis. However, *SOX9* alone is insufficient for cartilage formation; *SOX5* or *SOX6* is required for *SOX9* to drive chondrogenesis. The combination of *SOX5*, *SOX6*, and *SOX9*, often referred to as the *SOX* trio, provides sufficient signals for the formation of permanent cartilage.

*SOX9* is primarily expressed in chondrogenic mesenchymal cells, prechondrocytes, and chondrocytes, but it is not expressed in hypertrophic chondrocytes [19]. It is required for the commitment and differentiation of pluripotent mesenchymal cells toward chondrogenic lineages [20]. *SOX9* can also regulate chondrocyte proliferation and differentiation by directly controlling the expression of chondrocyte-specific genes. During embryonic cartilage development, mesenchymal progenitor cells exist as aggregates and condensation-like spheroids [21]. *SOX9* expression levels are elevated in condensing mesenchymal progenitors both in vitro and in vivo. The mesenchymal cells of *SOX9*-knockout mice cannot undergo condensation [22,23]. In *SOX9*-deficient mice, mesenchymal cells were absent from all cartilage but were present in juxtaposed mesenchyme that did not express chondrocyte markers such as c collagen and aggrecan [24]. Moreover, the conditional inactivation of *SOX9* in limb buds before mesenchymal condensation resulted in the complete absence of chondrocytes and conditional inactivation of *SOX9* after mesenchymal condensation, resulting in severe generalized chondrodysplasia [11].

*SOXs* are genes that encode transcription factors and regulate mRNA synthesis. *SOX9* is a transcription factor that influences the transcription of *SOX5* and *SOX6*. *SOX5* and *SOX6* share only 50% identity with SOX9 in the high-mobility group domain, and unlike *SOX9*, they do not feature a transactivation domain [25]. They not only are expressed with *SOX9* in prechondrocytes but also can enhance the transcriptional activity of *SOX9* [5]. Previous analyses have revealed that *SOX5* and *SOX6* bind to the same enhancers as *SOX9*, securing *SOX9* binding to DNA [25]. In vitro, *SOX5* and *SOX6* also bind with *SOX9* to chondrocyte-specific enhancers in *Col2a1*, *Acan*, and other chondrocyte genes, indicating that *SOX9* is a transcriptional activator required for chondrogenesis [26]. Mice with individual *SOX5^−/−^* or *SOX6^−/−^* variants are born with mild skeletal abnormalities, and mice with both *SOX5^−/−^* and *SOX6^−/−^* variants die in utero with rudimentary and poorly developed cartilage, expressing type II collagen and aggrecan at low-to-undetectable levels [27]. The expression of *SOX9* in *SOX5*^−/−^/*SOX6*^−/−^ mutant mice is insufficient for the formation of cartilage primordia [20]. *SOX5/6* homodimers and heterodimers can bind close to *SOX9* on cartilage-specific super enhancers. The forced expression of *SOX5/6* and *SOX9* is sufficient for the differentiation of stem cells into chondrocytes.

### 2.2. Runt-Related Transcription Factors (RUNX)

Runt-related transcription factor (RUNX) proteins, namely, RUNX1, RUNX2, and RUNX3, are a family of transcription factors expressed during chondrogenesis. *RUNX1* is involved in early chondrogenic differentiation and is also expressed by chondrocyte progenitors [28,29]. *RUNX2* generally modulates the progression of OA by suppressing chondrocyte hypertrophy. *RUNX3* regulates target genes during chondrocyte development and accelerates chondrocyte differentiation and maturation.

In vitro, *RUNX1* suppresses the hypertrophic differentiation of cultured chondrocytes. The chondrogenic compounds TD-198946 and kartogenin also facilitate chondrogenesis by inducing *RUNX1* expression [30]. *RUNX1* can enhance cartilage matrix production and induce chondrogenic transcription factors such as *SOX* genes. In addition, *RUNX1* activates the *COL2A1* promoter by binding to the *RUNX* motif of the 5′ flanking regions. In articular cartilage, *RUNX1* gene expression is decreased in patients with OA [30]. Aini et al. demonstrated that the intra-articular injection of polyplex nanomicelles containing *RUNX1* mRNA could suppress the development of surgically induced OA in mice [31]. 

Collectively, these data support the protective role of *RUNX1* in articular-cartilage maintenance; however, the molecular mechanisms underlying the enhancement of cartilage matrix production and suppression of hypertrophic differentiation by *RUNX1* remain poorly understood.

The role of *RUNX2* in skeletal development and OA has been thoroughly studied. The *RUNX2* protein is highly expressed in the prehypertrophic and hypertrophic zones of limb epiphyseal cartilage, increasing hypertrophic differentiation [32]. Kamekura et al. reported that *RUNX2* contributes to the pathogenesis of OA by inducing chondrocyte hypertrophy and matrix breakdown [33]. Numerous studies have reported that *RUNX2* deficiency decelerates the progression of OA by suppressing hypertrophic differentiation. In addition, *RUNX2* deletion causes a lack of ossification that impairs chondrocyte maturation [34]. Specific *RUNX2*-deficient chondrocytes partially attenuate the destabilization of the medial meniscus (DMM), a surgery-induced OA-like defect in adult mice [35].

*SOX9* primarily controls the regulation of *RUNX2*. This regulation involves CYPA, a positive regulator of *RUNX2* and *SOX9* expression in chondroprogenitor cells. In vitro, *CypA* knockdown suppresses chondrogenesis and endochondral ossification. *NKX3.2* serves as another regulator of *RUNX2*. *NKX3.2* represses *RUNX2* activity through direct interaction with the *RUNX2* promoter, and this repression is required for the progression of bone morphogenetic protein–induced chondrogenesis. *COL10A1*, a type of collagen expressed in hypertrophic chondrocytes destined for endochondral ossification, is also a direct transcriptional target of *RUNX2*. Hinoi et al. demonstrated that *RUNX2* enhances the expression of *FGF18*, a negative regulator of chondrocyte maturation, in the perichondrium [36]. Thus, *RUNX2* is generally regarded as the dominant regulator of chondrocyte hypertrophy.

*RUNX2* has another potent function in inducing osteogenesis. The regulation of osteogenesis and chondrocyte proliferation by *RUNX2* is accomplished through interaction with PI3K–Akt signaling. In addition to inducing osteogenesis, *RUNX2* has also been shown to increase adipogenic differentiation in vitro.

*RUNX3* plays a key role in chondrocyte differentiation. The expression of *RUNX3* is increased in prehypertrophic chondrocytes and maintained in hypertrophic chondrocytes. However, *RUNX3* is reduced in terminal hypertrophic chondrocytes, and the *RUNX3* expression pattern overlaps with that of *RUNX2* [37]. At a transcriptional level, *RUNX2* and myocyte enhancer factor-2C (*MEF2C*) have been implicated as key transcription factors regulating chondrocyte hypertrophy, as they drive the expression of terminal differentiation markers, type X collagen, MMP3, MMP13, integrin-binding sialoprotein (IBSP), Indian hedgehog (Ihh), and alkaline phosphatase [38]. In humans, *MEF2C* and *RUNX3*, but not *RUNX2*, are the key transcription factors driving hypertrophy and regulating the endochondral pathway in MSCs [39]. These phenomena suggest that *RUNX3* is key for the formation of hypertrophy-resistant cartilage because it facilitates the optimal utilization of MSCs between the chondral and endochondral pathways.

### 2.3. Trichorhinophalangeal Syndrome Type 1 (TRPS1)

Trichorhinophalangeal syndrome type 1 (*TRPS1*) is a chondrogenic GATA-like transcription factor that serves as a regulator of chondrocyte differentiation and proliferation. Suemoto et al. demonstrated that *TRPS1* regulates chondrocyte proliferation and survival by controlling *STAT3* expression. *TRPS1* inhibits *STAT3* expression, which controls chondrocyte proliferation and survival by regulating the expression of *CYCLIN* *D1* and *BCL2*. In addition, Trps1 interacts with Ihh/Gli3 signaling and affects chondrocyte differentiation and proliferation [40]. A disrupted *Trps1* gene in mice also results in chondrodysplasia due to the reduced proliferation of chondrocytes and decreased apoptosis in growth plates [41]. In *Trps1*-knockout mice, parathyroid-hormone-related protein is overexpressed and Ihh-mediated chondrogenesis is inhibited through an Ihh/PTHrP negative-feedback loop [42].

*TRPS1* interacts with many miRNAs and other signaling pathways. *TRPS1* can maintain the low level of miR-221 that allows MSCs to differentiate into the chondrocyte lineage [43]. The interaction between *TRPS1* and miR-221 could be a feedback loop, and *TRPS1* expression is regulated by seven *RUNX2*-targeting miRNAs: miR-23a, miR-30c, miR-34c, miR-133a, miR-135a, miR-205, and miR-217 [44]. The essential transcription factors that regulate the chondrogenic differentiation of MSCs and the factors’ respective miRNAs are listed in Table 1.

## 3. Negative Regulators of Chondrogenesis

Chondrogenesis is not only positively regulated by SOX and other proteins but also negatively regulated by some transcription factors, which inhibit the expression of ECM proteins in chondrogenic cells. The negative regulators Slug and C/EBPβ are expressed by hypertrophic chondrocytes, which indicates that they control terminal chondrocyte differentiation and promote endochondral ossification [58] (Figure 2). *δEF1*, *AP-2α*, and *Twist1* are expressed by chondrocyte progenitors and prevent the production of cartilage-like mesenchymal tissue, indicating that they negatively regulate early phase chondrogenesis [59,60,61,62,63].

## 4. MSCs Derived from Synovial Tissue Have Strong Chondrogenic Potential

Because of their ease to be harvested, high proliferation rate and differentiation capacity, and strong resistance to the effects of age and passage, ADSCs, particularly those isolated from subcutaneous adipose tissue (SC-ADSCs), may be effective candidates for cell-based regenerative therapies, especially for older adult patients [64]. SC-ADSCs are candidate MSCs for the treatment of OA because they promote chondrogenesis and inhibit the inflammation of cartilage.

In addition to SC-ADSCs, synovium-derived MSCs (SDMSCs), which are derived from the synovial membrane surrounding joints, exhibit stronger chondrogenic potential both in vitro and in certain in vivo states. The synovium is a postnatal reservoir of MSCs. The synovium descends from the embryonic joint interzone and maintains joint tissues in adults. Roelofs et al. discovered that human SDMSCs exhibit morphogenetic properties by patterning a joint-like organ in vivo [65]. When researchers compared synovium and cartilage with respect to the anatomical location and functional structure, SDMSCs were determined to possess a tissue-specific nature and to have a gene expression profile similar to that of chondrocytes. Therefore, SDMSCs respond most appropriately to signaling in the joint cavity, thereby, facilitating cartilage regeneration [66].

Two subsets of SDMSCs can be obtained. Fibrous SDMSCs can be harvested from the noncartilaginous area of the lateral condyle of the femur, an area that is overlaid with the lateral part of the knee joint capsule. Adipose SDMSCs are located in the IPFP behind the patellar tendon. Cells derived from fibrous and adipose synovium exhibit higher proliferative potential, colony-forming efficiency, and cartilage matrix production than do those derived from subcutaneous fat. Although both SDMSCs are superior in terms of their chondrogenic potential, the properties of fibrous SDMSCs are more favorable for cartilage formation [67]. Inflammatory activity in the synovium changes the composition and functional characteristics of SDMSCs [68]. Although adipose SDMSCs do not possess all the favorable qualities of fibrous SDMSCs, they may still be a superior choice for the treatment of OA because few inflammatory changes are associated with the IPFP [66].

## 5. IPFP-MSCs Are Superior to Other MSCs in OA Treatment

MSC therapies have exhibited the potential to regenerate cartilage in animal and preclinical studies [69]. The initial proposal of using MSCs for cartilage repair was based on the ability of MSCs to differentiate into chondrocytes to directly replace damaged cartilage. Because the proliferation and differentiation capacities of SC-ADSCs are minimally affected by age and multiple passages, SC-ADSCs may be effective candidates for cell-based regenerative therapies, especially for older adult patients [64]. SC-ADSCs are candidate MSCs for the treatment of OA because they promote chondrogenesis and inhibit the inflammation of cartilage.

The IPFP is located intra-articularly and extrasynovially in the knee joint. It is primarily located between the joint capsule and the synovial membrane. It relieves shock to the knee and protects the knee joint under physiological conditions or in the early stage of knee OA. Therefore, the role of the IPFP in degenerative arthritis has attracted increasing attention.

An orthopedic surgeon can easily collect IPFP tissue during high tibial osteotomy (HTO), total knee replacement (TKR), or knee arthroscopy. Additionally, the use of IPFP tissue eliminates the need to collect subcutaneous fat tissue from other sites, which can effectively reduce the number of additional surgical procedures required. Moreover, IPFP-MSCs can serve as a source for establishing an allogenic cell bank for OA therapies in the future.

The IPFP may secrete factors that protect the knee joint, such as lipid-mediated lipoxin A4. The levels of lipid-mediated lipoxin A4 in the IPFPs of patients with OA are higher than those in the IPFPs of healthy individuals, and the IPFP can prevent cartilage degradation in the knee [70,71]. The IPFP also secretes leptin, which can promote the production of articular-cartilage proteoglycan and type II collagen and stimulates the synthesis of growth factors (namely, insulin-like growth factor-1 and transforming growth factor-β), thereby, enhancing chondrocyte proliferation and protecting against the pathogenesis of knee OA [72,73]. The IPFP can also block the secretion of proinflammatory mediators in the synovia and chondrocytes of patients with OA [9]. 

In addition to the aforementioned advantages, the chondrogenic capacity of IPFP-MSCs is comparable to that of SDSCs, with no significant differences between the capacity between young and older adult donors. IPFP-MSCs are readily available to orthopedic surgeons and can resist inflammation and senescence, rendering them superior to SDSCs and SC-ADSCs for use in the treatment of knee OA. The chondrogenic capacity of IPFP-MSCs is greater than that of bone-marrow-derived MSCs (BMSCs) and subcutaneous ADSCs [74]. Chang et al. reported that patients with knee OA who received a single intra-articular injection of autologous IPFP-MSCs exhibited suppressed inflammation and alleviation of disease symptoms [75].

Manferdini et al. demonstrated the anti-inflammatory effects of downregulating the expression of inflammatory factors (such as IL-1β, IL-6, and IL-8) produced by chondrocytes or synoviocytes during coculture [9]. Koh et al. also demonstrated that the intra-articular injection of a combination of IPFP-MSCs and platelet-rich plasma effectively reduced pain and improved knee function in patients with knee OA [76]. This indicates that IPFSCs, in addition to SDSCs, may serve as a cell source for cartilage regeneration [77]. These findings also elucidate how the function of IPFP-MSC-based therapies and the immunomodulatory properties of MSCs make them ideal for treating peri-articular and intra-articular pathologies associated with OA.

## 6. MSC Delivery Mechanisms

MSCs can be delivered using a hydrogel carrier, by direct injection, or through magnetic fields. Injectable stem cell carriers, including hyaluronic acid (HA) and various hydrogel systems [78], are used in translational and clinical applications. HA is commonly used to deliver MSCs to treat cartilage lesions and joint degeneration. HA exhibits excellent biocompatibility with MSC cells. Desando et al. reported that the combination of MSCs and HA regulated cell homing while promoting attachment and integration within the damaged articular cartilage [79]. Huang et al. determined that IPFP-MSCs combined with chitosan/hyaluronic-acid nanoparticles promoted chondrogenic differentiation [80]. Moreover, agarose hydrogels combined with porcine IPFP-MSCs increased chondrogenic differentiation in vitro and mechanical functionality [81]. Fibrin hydrogels incorporated with TGF-β1-loaded gelatin microspheres in porcine IPFP-MSCs also enhanced in vitro chondrogenesis by enhancing glycosaminoglycan production [82]. Sekiya et al. reported that eight patients with OA who received synovial MSC injections without carriers exhibited significant inhibition of alterations in the projected cartilage area ratio in the femoral posteromedial region and improvement of clinical scores at 30 weeks after injection. In addition, Ochi et al. reported that delivering magnetically labeled MSCs (m-MSCs) by using magnetic fields was a potential approach to repairing cartilage defects and had no adverse effect on chondrogenic differentiation [83].

## 7. Ethical and Regulatory Points for Developing Autologous and Allogeneic MSC Products

IPFP-MSCs have several advantages over other types of MSCs for cartilage regeneration. IPFP tissue can be quickly harvested during concomitant orthopedic surgery without bone-marrow aspiration. Although the synovium can also be harvested during concomitant orthopedic surgery, the volume of the synovium is lesser than that of the IPFP.

The regulation of cell-therapy development to protect donors’ safety and reduce costs is essential. In Japan, the Pharmaceuticals and Medical Devices (PMD) Act and the Act on the Safety of Regenerative Medicine (ASRM) were established in November 2014. The PMD Act defines regenerative medical products and introduces a system for the conditional and time-limited authorization of marketing of regenerative medical products [84]. For the regulation of cell-therapy programs, the government of Taiwan announced the Regulations of Special Medical Techniques, mainly permitting autologous cell-therapy programs, in September 2018.

Many autologous and allogeneic MSC products have been approved, including the implantation of autologous chondrocytes (ACIs) and matrix-induced autologous chondrocyte implantation (MACI), a technology derived from simple ACI that comprises a two-step arthroscopic procedure that is effective in young patients with focal injuries [85]. CARTISTEM, a combination of human allogenic umbilical cord blood–derived MSCs and sodium hyaluronate, is intended to be used as a single-dose cellular therapeutic agent for cartilage regeneration in patients with cartilage defects [86]. DeNovo NT Graft is an FDA-listed tissue product and a particulate juvenile cartilage allogenic implant used to repair articular-cartilage damage. It provides surgeons with an option for early intervention for articular-cartilage repair [87]. The increasing number of cell products based on autologous and allogeneic MSCs should facilitate the individualization of regenerative therapies.

Allogeneic MSCs are frequently offered worldwide as a universal human remedy. However, undesired differentiation and malignant transformation are major safety issues regarding the transplantation of allogeneic cells. Protocols for the differentiation of MSCs should ensure an allogeneic source tissue, from which MSCs can be isolated safely and efficiently for clinical use. Because allogeneic MSCs may promote tumor growth and metastasis, studies utilizing MSCs should focus on the continuous monitoring and long-term follow up of MSC-treated animal models to determine possible protumorigenic and other detrimental effects of MSC-based therapies [88].

## 8. The Potential of MSC-Secreted Exosomes

MSCs clearly have therapeutic potential for cartilage repair. However, the sources of MSCs have limitations, and because of variations in donor age and restricted proliferation capacity during in vitro expansion, chondrogenesis is not easy to maintain. Therefore, the need for strict control of MSC isolation, collection, storage, and transportation limits the efficacy of MSC therapies. Moreover, the use of live cells may result in inevitable safety risks such as immune rejection, tumorigenesis due to uncontrolled cell differentiation, and the inability to remove transplanted cells in the case of adverse reactions.

An increasing amount of evidence has indicated that the paracrine effect of MSCs also stimulates cartilage repair [89]. The therapeutic effects of MSCs are mediated by the secretion of essential functional soluble factors present in the EVs released by MSCs. Depending on their size and biogenesis pathways, EVs can be classified into exosomes (30–150 nm), microvesicles (MVs; 100–1000 nm), or apoptotic bodies (1000–5000 nm) [90]. MSC-secreted exosomes are the most commonly studied. Compared with other EVs, MSC-secreted exosomes are more stable under various physiological conditions and are immune-privileged to a certain degree. Thus, MSC-derived exosomes are suitable for therapeutic applications, and they have become popular as a cell-free MSC therapy in the field of cartilage engineering [91]. Exosomes derived from human embryonic MSCs have been reported to stimulate cartilage regeneration [92]; in addition, exosomes derived from BMSCs protect cartilage from degeneration in vivo and in vitro [93]. Moreover, IPFP-MSC-derived exosomes exhibit greater potential for the treatment of OA than do other MSCs; therefore, they should be further optimized for clinical applications.

### 8.1. Effects of Exosomes from Different MSCs

EVs can deliver specialized messenger molecules as biological signals. The compositions of these molecules are highly dependent on specific conditions. The molecules packed within EVs not only represent the desired messages that contribute to therapy but also include undesirable messages that facilitate the spread of disease [94]. For example, Domenis et al. determined that exosomes derived from OA synovial joint fluid could activate inflammatory cells and stimulate the release of inflammation-related cytokines, chemokines, and metalloproteinases by M1 macrophages [95]. Additional exosome functions have been observed in OA [96]. Therefore, the packaging of exosome content by the secreting cell is programmable and subject to a regulatory mechanism. Exosomes secreted from different unmodified MSCs or conditioned medium-treated MSCs have been reported to have therapeutic value for patients with OA [97].

Exosomes from different cell types may exert different effects on OA, but these effects are still under investigation. Exosomes derived from chondrocytes (CC-Exos) stimulate the proliferation of cartilage progenitor cells and significantly promote the expression of chondrogenesis-related factors. They increase collagen deposition, minimize vascular ingrowth, and efficiently and reproducibly develop into cartilage [98]. Exosomes from BMSCs (BMSC-Exos) repaired cartilage damage in rats with OA by carrying high amounts of miR-135b, thereby, targeting proinflammatory factors upregulated in the serum of rats [99]. Moreover, BMSC-Exos inhibit chondrocyte apoptosis and MMP expression by regulating Drp1-mediated mitophagy [100]. Exosomes from embryonic MSCs (EMSC-Exos) could maintain the chondrocyte phenotype by promoting collagen type II synthesis and decreasing ADAMTS5 expression. EMSC-Exos may contribute to the adenosine-mediated activation of protein kinases, transforming growth factor-β (TGF-β), and insulin growth factor (IGF) [101].

Human synovial MSC–derived exosomes (hSMSC-Exos) stimulate chondrocyte proliferation and migration by inducing the overexpression of WNT5A, leading to the activation of YAP signaling pathways and, ultimately, the suppression of ECM formation [102,103].

Exosomes from SC-ADSCs (ADSC-Exos) may play a chondroprotective role by downregulating senescence-associated β-galactosidase activity as well as reducing the production of inflammatory and catabolic mediators from OA osteoblasts and OA chondrocytes, respectively [104]. Shao demonstrated that chondrocytes treated with exosomes isolated from IPFP-MSCs (IPFP-Exos) exhibited higher SOX-9, aggrecan, and Col II expression and higher performance than did exosomes secreted from IPFP-MSCs pretreated with kartogenin [105].

### 8.2. Paracrine Role of MSC-Secreted Exosomes in Chondrogenesis

The effects of MSC-based therapies and the chondrogenic potential of MSCs are often attributed to paracrine secretion, particularly the secretion of exosomes. Exosomes have biological functions similar to those of parental cells. Most studies have used MSCs as sources of exosomes because of their major therapeutic benefits for tissue repair and regeneration. Therefore, the interest in exploiting exosomes to mediate cartilage regeneration is growing [106].

Exosomes function primarily as intercellular communication vehicles to transfer bioactive lipids, nucleic acids, and proteins from donor cells into recipient cells to obtain biological responses [89]. Exosomes that mediate the paracrine effect of MSCs effectively protect cartilage [107]. These biological responses translate to therapeutic outcomes in injured cartilage or in the microenvironments of tissues affected by MSC-derived exosomes. Exosome therapy can protect articular chondrocytes from apoptosis induced by H_2_O_2_. Exosomes isolated from ADSCs suppress the expression of IL-6, NF-κB, and TNF-α and promote the expression of IL-10. In addition, exosome therapy promotes the chondrogenesis of periosteal cells and increases type II collagen and β-catenin levels [108].

### 8.3. MSC Exosomal MiRNA Therapy in Cartilage Protection

**Figure 3:**
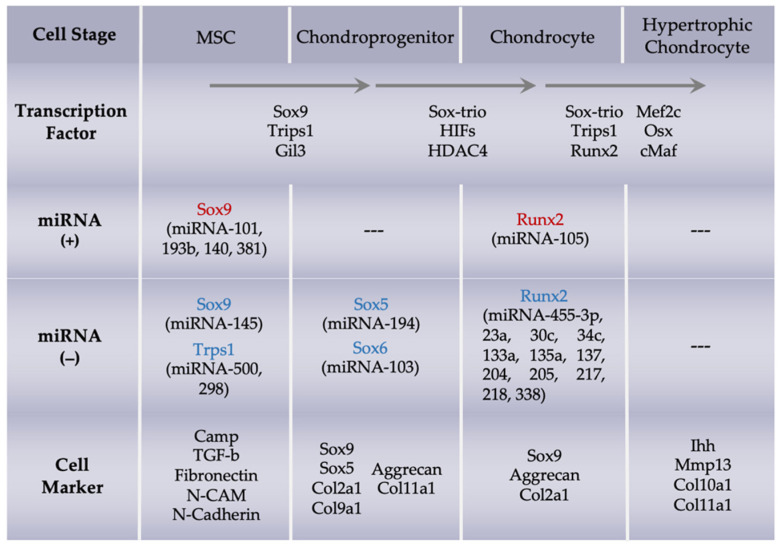
Schematic illustrating transcription factors and miRNA regulators involved in chondrocyte differentiation of adipose-derived stem cells (ADSCs). Abbreviations: sex-determining region Y–related high-mobility group box 9 (SOX9); trichorhinophalangeal syndrome type 1 (TRPS1); runt-related transcription factor 2 (RUNX2); hypoxia inducible factor (HIF); histone deacetylase 4 (HDAC4); myocyte enhancer factor 2c (MEF2c); osterix (Osx); C-musculoaponeurotic fibrosarcoma (cMaf); cathelicidin antimicrobial peptide (CAMP); transforming growth factor beta (TGF-β); neural cell adhesion molecule (N-CAM); type II procollagen (Col2a1); type IX collagen alpha 1 chain (Col9a1); type XI collagen (Col11a1); Indian hedgehog (Ihh); matrix metalloproteinase 13 (MMP13); type X collagen (Col10a1).

Exosomes serve as carriers of various biomolecules, including DNA, mRNA, miRNAs, proteins, and lipids, between cells, thereby, modulating biological processes and contributing to cell–cell communication as well as influencing the progression of various diseases. Transcription factors are regulated by the expression of miRNAs and cellular markers, as indicated in Figure 3.

An analysis of periosteal cells treated with exosomes revealed that high levels of miR-145 and miR-221 are related to the enhanced proliferation of periosteal cells and chondrogenic potential, respectively [108]. MiR-100-5p derived from IPFP-MSCs can significantly enhance the autophagy of chondrocytes through mTOR inhibition. The intra-articular injection of antagomir-100-5p protected cartilage and ameliorated the gait patterns of mice with DMM-induced OA by inhibiting chondrocyte apoptosis through the mTOR–autophagy pathway [109]. Exosomes derived from miR-140-5p-overexpressing human synovial MSCs could stimulate cartilage regeneration and delay knee OA progression in a rat OA model [110]. Exosomes derived from human miR-92a-3p-overexpressing BMSCs inhibited cartilage degradation in a collagenase-induced OA mouse model by directly targeting WNT5A and maintaining the function of articular chondrocytes [111]. In addition, TUG1 modulates chondrocyte proliferation, apoptosis, and IL-1β-induced ECM degradation in human cartilage C28/I2 cells via the miR-320c/FUT4 axis. The upregulation of TUG1 in OA tissues is modulated by miR-320 [112]. In a rat OA model, TGF-β1 promoted chondrocyte proliferation by regulating Sp1 through BMSC-exosome-derived miR-135b and promoted cartilage repair [113] (Table 2). Taken together, these results indicate that miRNA regulation is involved in modulating gene expression during chondrogenic differentiation. The validation of these miRNAs and their targets may facilitate further research on safe and efficient novel delivery systems that may promote the use of miRNAs in OA therapy.

Chondrogenic differentiation is initiated by the chondrogenic lineage commitment and MSC condensation, proliferation, differentiation (into chondrocytes), and terminal differentiation (into hypertrophic chondrocytes) processes, which is associated with the expression of cartilage-specific genes. ADSCs from the IPFP play a key role in cartilage generation; therefore, investigation and regulation of the chondrogenic differentiation of IPFP-MSCs are necessary to understand and treat degenerative cartilage diseases. In addition, miRNAs are involved in the cartilage differentiation of MSCs and play a pivotal role in cartilage regeneration. Figure 3 summarizes the transcription factors, miRNAs, and markers involved in the chondrogenesis of MSCs.

## 9. Conclusions

In this review, we discussed the key role of IPFP-MSCs in the disease progression of knee OA and IPFP-MSCs’ strict regulation by transcription factors during chondrogenesis. We also explored the potential applications of exosomes in the treatment of OA because obtaining human IPFP-MSCs from patients with OA through arthroscopic operation is convenient and feasible. In addition, the differentiation of IPFP-MSCs into the chondrocyte lineage can be genetically manipulated and is promoted by specific transcription factors. Studies have explored the integration of transcription factors, including SOX family members, RUNX family members, and Trps1, in the differentiation of MSCs. The overexpression of single or multiple transcription factors in IPFP-MSCs may promote differentiation into the chondrocyte lineage, which can be used for cartilage regeneration.

Exosomes have been discovered to have therapeutic value in the treatment of OA. Exosomes have a relatively long lifespan and can be stably stored at low temperatures for a long time. In addition, exosomes can deliver nucleic acid and protein drugs to target cells and protect the target cells from enzymatic degradation. Therefore, exosomes can be modified to carry specific medicines to meet the needs of particular treatment regimens. To address the safety concerns associated with the limited chondrogenic function of MSCs isolated from older adult donors, experts have generally studied the applications of exosomes in cartilage repair. Herein, we summarized the present studies of exosomes in direct and indirect potential therapeutic strategies for OA, emphasizing the roles of IPFP-MSC-derived exosomes containing miRNAs. IPFP-MSC exosomes also exerted a strong stimulatory effect on chondrocyte proliferation and differentiation. IPFP-MSC exosomes may represent a novel therapeutic approach for OA treatment in future clinical settings.

## Figures and Tables

**Table 1:** MicroRNAs involved in transcription factor regulation during chondrogenesis.

miRNA	Transcription Factor	Effect on Chondrogenesis	Cell Type/Condition	Study Model	Reference
miR-101	*Sox9*	(+)	Rat BMSCs	miR-101 increases expression of *Sox9* and decreases expression of *Runx2*.	[45]
miR-193b	*SOX9*	(+)	Human Chondrocytes	Maintenance of chondrocytes and ECM homeostasis.	[46]
miR-140	*Sox9*	(+)	Mouse Embryo	miR-140 proximal promoter activity and miR-140 expression are upregulated by *Sox9* in chondrocytes. miR-140 was identified as a cartilage-specific microRNA that could be a critical regulator of cartilage development and homeostasis in genetically modified mice.	[47]
miR-381	*Sox9*	(+)	Mouse Chondrocytes	miR-381 is highly expressed during chondrogenesis and in arthritic cartilage. *Runx2* and *Sox9* upregulate expression of miR-381.	[48]
miR-101	*Sox9*	(−)	Rat Chondrocytes	miR-101 participates in IL-1β-induced chondrocyte ECM degradation.	[49]
miR-145	*Sox9*	(−)	Murine BMSCs	miR-145 is a key negative regulator of chondrogenic differentiation that directly targets *Sox9* at early stages of chondrogenic differentiation.	[50]
miR-145	*SOX9*	(−)	Human Chondrocytes	miR-145 negatively regulates endogenous *SOX9* expression in human articular chondrocytes.	[51]
miR-194	*SOX5*	(−)	Human ADSCs	MiR-194 inhibits *SOX5* expression and inhibits early chondrogenic differentiation.	[52]
miR-103	*SOX6*	(−)	Human Primary Chondrocytes	miR-103 promotion of osteoarthritis is mediated by downregulation of *SOX6*.	[53]
miR-455, miR-210	*RUNX1*	(+)	Human Synoviocytes	*RUNX1* can bind to the promoter regions of miR-455 and miR-210 and protects the synovium against cartilage degeneration.	[54]
miR-105	*RUNX2*	(+)	Human Primary Chondrocytes	*RUNX2*, a key transcription factor involved in OA progression, has been identified as a direct target of miR-105.	[55]
A panel of miRNAs (miR-23a, -30c, -34c, -133a, -135a, -137, -204, -205, -217, -218, -338)	*Runx2*	(+)	Mouse Chondroprogenitor Cell Line ATDC5	Promotion of early chondrogenic differentiation and suppression of chondrocyte maturation.	[56]
miR-455-3p	*Runx2*	(−)	Mouse Chondroprogenitor Cell Line ATDC5	MiR-455-3p induces early chondrogenesis by inhibiting *Runx2*.	[57]
-	*Runx3*	-	-	-	-
miR-500, miR-298	*Trps1*	(−)	Mouse Chondroprogenitor Cell Line ATDC5	*Trps1* inhibits the expression of miR-500 and miR-298, which controls the chondrocyte hypertrophy transcription factor *Mef2c*.	[39]

**Table 2:** MicroRNAs involved in cartilage regeneration.

miRNA	Cell	Source	Species	Mechanism	Function	Reference
miR-100-5p	ADSC	IPFP	Human	MiR-100-5p promotes autophagy of chondrocytes through mTOR inhibition.	MiR-100-5p protects cartilage from damage in mice with destabilization of the medial meniscus (DMM) surgery-induced OA.	[109]
miR-140-5p	SMSC	Synovial Membrane	Human	SMSC-Exos activate yes-associated proteins, decreases ECM secretion, and induces proliferation and migration of articular chondrocytes via WNT5A and WNT5B.	SMSC-140-Exos enhance the proliferation and migration of articular chondrocytes without damaging ECM secretion in vitro; in vivo, SMSC-140-Exos successfully prevented OA in a rat model.	[110]
miR-92-3p	BMSC	Bone Marrow	Human	Exosomal miR-92a-3p regulates cartilage development and homeostasis by directly targeting WNT5A.	MSC-miR-92a-3p-Exos inhibited cartilage degradation in an OA mouse model.	[111]
miR-320c	BMSC	Bone Marrow	Human	TUG1 modulates chondrocyte proliferation and apoptosis and ECM degradation in IL-1β-induced C28/I2 cells via the miR-320c/FUT4 axis.	miR-320c upregulates TUG1 in OA tissues and is modulated by miR-320.	[112]
miR-135b	BMSC	Bone Marrow	Rat	TGF-β1 promotes chondrocyte proliferation by regulating Sp1 through MSC-exosome-derived miR-135b and then promotes cartilage repair.	TGF-β1 promotes cartilage repair by regulating Sp1 through miR-135b in vivo.	[113]

Abbreviations: synovial mesenchymal stem cells (SMSCs); adipose-derived mesenchymal stem cells (ADSCs); bone-marrow-derived mesenchymal stem cells (BMSCs); exosomes (Exos).

## Data Availability

Not applicable.
